# Gender differences in the association of insulin resistance and high-sensitivity c-reactive protein in obese adolescents

**DOI:** 10.1186/2251-6581-13-35

**Published:** 2014-02-20

**Authors:** Ramin Alemzadeh, Jessica Kichler

**Affiliations:** 1 Department of Pediatrics, University of Illinois at Chicago, Chicago, IL, USA; 2 Behavioral Medicine and Clinical Psychology, Cincinnati Children’s Hospital Medical Center, Cincinnati, OH, USA

**Keywords:** Metabolic syndrome, Diabetes, c-reactive protein, Obesity, Dyslipidemia

## Abstract

**Background:**

Low-grade vascular inflammation is believed to initiate early atherosclerotic process by inducing insulin resistance (IR), with significant gender differences in adults. We evaluated the relationship between surrogate measures of inflammation and IR in obese adolescents.

**Methods:**

The association among markers of inflammation [high-sensitivity c-reactive protein (hs-CRP)] and IR, cardiometabolic risk factors and body composition was retrospectively examined in 199 obese adolescents [(111 F/88 M), aged 15.5 ± 1.2 years]. Insulin resistance was assessed using homeostatic model assessment for insulin resistance (HOMA-IR).

**Results:**

Males had higher body mass index SD-score (BMI-SDS), fat mass (FM), glucose, insulin, HOMA-IR, HbA_1c_, hs-CRP, triglycerides: HDL-C (TG:HDL-C) ratio than females (p < 0.05), whereas females had higher c-peptide: insulin ratio than males (p < 0.05). Also, 50.8% of subjects were identified with metabolic syndrome with similar gender distribution (M: 57.9% vs. F: 45.1%, p = 0.32). Hs-CRP was correlated with HOMA-IR in the cohort, even when controlling for FM (*r* = 0.26; p < 0.0001). However, hs-CRP and HOMA-IR displayed a significant correlation only in females (*r* = 0.37; p < 0.0001) when adjusting for FM and pubertal status. Also, c-peptide: insulin ratio was inversely correlated with hs-CRP (*r* = −0.32; p < 0.001) and HOMA-IR (*r* = −0.62; p < 0.0001) and partially mediated the relationship between these biomarkers only among females (β = 0.36, p < 0.001 to β = 0.18, p < 0.05; Sobel Test: p < 0.01).

**Conclusions:**

A positive association between hs-CRP and HOMA-IR was observed only in adolescent girls which was influenced by altered hepatic insulin clearance. This implies that obese adolescent girls may be at greatest risk of developing early atherosclerosis and diabetes.

## Introduction

Metabolic syndrome (MS) is a collection of metabolic abnormalities that include visceral obesity, insulin resistance (IR), hypertension, hypertriglyceridemia, a low level of high-density lipoprotein cholesterol (HDL-C) and hyperglycemia in adults and adolescents [1,2]. In individuals with MS, IR plays a major role in the pathogenesis of type 2 diabetes mellitus (T2DM) and cardiovascular diseases [3,4]. An IR state is associated with low grade inflammatory response characterized by abnormal production of cytokines and the activations of pro-thrombotic and pro-inflammatory signaling pathways which is associated with an increased risk of T2DM, myocardial infarction and stroke in adults [5-8].

Adipose tissue secretes a number of inflammatory cytokines, such as interleukin-6 (IL-6), which induces hepatic production of c-reactive protein (CRP), a known systemic inflammatory biomarker. This systemic inflammatory marker is also called high-sensitivity CRP (hs-CRP) when measured in the serum with a high-sensitivity assay [9]. Indeed, several studies have shown that increased body adiposity among children and adults is associated with higher serum hs-CRP concentrations compared to normal-weight individuals [10-12]. Also, obesity is associated with a pro-inflammatory and pro-thrombotic state without known MS comorbidities in children, even prior to the onset of puberty [13].

A strong association between hs-CRP and cardiovascular risk independent of other established risk factors, such as dyslipidemia, blood pressure, alcohol consumption and tobacco smoking in adults has been observed [14,15]. Similarly, circulating hs-CRP has been shown to be an independent predictor of myocardial infarction, ischemic stroke, T2DM and hypertension in adults [15,16]. Also, elevated circulating hs-CRP level has been found to correlate with early vascular endothelial dysfunction among children and adolescents [17,18] suggesting that inflammation plays a major role in the pathogenesis of early atherosclerosis and likely begins in childhood with progression slowly into adulthood [19,20].

Previous studies have shown that women have higher hs-CRP levels than men, and this gender difference is maintained across all ethnic groups [21-23]. While exact mechanisms for this hs-CRP trend are not known, it is possible that the gender difference in the relationship between hs-CRP and obesity is due to relatively higher degree of adiposity in women compared to men [22]. Specifically, the quantity and distribution of body fat appear to influence hs-CRP levels to a greater extent in women compared to men [24]. Also, all components of MS may be more strongly associated with hs-CRP in women compared to men suggesting that inflammatory processes may play a strong role in the pathogenesis of MS in women [25]. Thus, we hypothesized that this gender difference in relationship between hs-CRP and MS and its components is already present during adolescence; and examined the association among markers of inflammation, insulin resistance and insulin clearance and adiposity in a group of obese adolescents.

### Subjects and methods

#### Subjects and design

One hundred and ninety-nine adolescents (age: 13.2-19.9 years) who met the criteria for obesity [body mass index (BMI) > 95^th^ percentile for age] [26] were included in the study. Subjects were evaluated at the Children’s Hospital of Wisconsin (CHW) Endocrine Clinic for evaluation of MS between February 2006 and October 2009. Race/ethnicity was self-assigned: Caucasian (C, n = 87; 43.7%), Mexican American [Hispanic (H), n = 60; 30.2%], and African American (AA, n = 52; 26.1%). Children were excluded if they had hepatic or renal disease, metabolic rickets, malabsorptive disorders (e.g., Crohn’s disease, cystic fibrosis, and/or celiac disease) or cancer, or were taking multivitamin supplements, anticonvulsants, or systemic glucocorticoids. In compliance with the 1964 Declaration of Helsinki, the CHW Institutional Review Board (IRB) approved the retrospective review of patients’ clinical charts; thus, informed consent was not required.

Data were collected on patients including age, gender, ethnicity, height, weight, blood pressure and body composition analysis by bioelectrical impedance (TANITA-TBF-410, TANITA Corporation of America Inc., Arlington Heights, IL) for evaluation of fat mass (FM), fat-free mass (FFM) and total body water (TBW) [27,28]. Two well-trained clinicians determined pubertal maturation (Tanner stage). Fasting serum samples were obtained for glucose, insulin, c-peptide, hemoglobin A_1c_ (HbA_1c_), lipid profiles and hs-CRP.

#### Laboratory studies and calculations

All blood samples were obtained between 0800 and 1100 h after an overnight fast. Serum glucose was measured by an autoanalyzer (Orthodiagnostics Fusion 5.1, Ortho-Diagnostics, Rochester, NY). The hs-CRP assays were carried out at Quest Diagnostics (San Jose, CA) using a polystyrene particle-enhanced immunonephelometric method (Dade Behring BNII). The detection limit of this assay was 0.20 mg/L with measuring range of 0.18 to 1150 mg/L with intra-assay and inter-assay coefficients of variance of 2.65% and 3.6%, respectively. The hs-CRP values >10 mg/L were excluded to avoid influence of acute infection [29]. Hemoglobin A_1c_ (HbA_1c_) level was determined by the Bayer DCA (Bayer Diagnostics Inc, Tarrytown, NY) 2000 instrument (non-diabetic range of 4.5% to 5.7%).

Fasting serum insulin was measured by Nichols radio-immunoassay (RIA) (Nichols Institute, San Clemente, CA) with intra-assay and inter-assay coefficients of variation (CV) of 2.4-6.3% and 5.2-13.0%, respectively. The homeostatic model assessment estimates for insulin resistance (HOMA-IR) calculated as previously described [30]: HOMA-IR = (blood glucose mmol/L × insulin μU/mL)/22.5. Fasting serum c-peptide was measured by Nichols radio-immunoassay (RIA) (Nichols Institute, San Clemente, CA) with intra-assay and inter-assay coefficients of variation (CV) of 2.3-6.9% and 8.0-17.6%, respectively. Serum c-peptide: insulin ratio was calculated as a surrogate marker of hepatic insulin clearance.

Total cholesterol, high-density lipoprotein Cholesterol (HDL-C) and triglycerides (TG) were determined by colorimetric methods (Beckman spectrophotometer, Fullerton, CA). Low-density lipoprotein cholesterol (LDL-C) was calculated using Friedewald’s equation [31].

Blood pressure (BP) measurements were taken twice and averaged with the patient in sitting position. Elevated systolic or diastolic blood pressure (SBP or DBP) was defined as a value above the 95^th^ percentile for age, gender and height [32].

Modified National Cholesterol Education Program (NCEP) criteria [2] for the diagnosis of MS were defined as the presence of 3 or more of the following: age-adjusted BMI >95^th^ percentile, age-adjusted systolic or diastolic BP > 90^th^ percentile, age-adjusted TG >90th percentile, age-adjusted HDL cholesterol <5^th^ percentile, and impaired fasting glucose >5.6 mmol/L.

#### Statistical analysis

Statistical analyses were carried out using SPSS (version 14.0). Data are expressed as mean ± SD. Body mass index (BMI) values were converted into standard deviation scores (SDS), using 2000 Center for Disease Control (CDC) growth charts. The natural logarithmic transformation of the variables was used in the correlation and regression analyses when they were found to be skewed. Differences between those with MS versus those who did not meet the MS criteria (non-MS) were compared using unpaired student *t* tests. The differences among ethnic subgroups were evaluated by one-way analysis of variance (ANOVA), and Bonferroni’s post-hoc testing was applied whenever appropriate. Chi-square analyses were used to compare prevalence of MS. Spearman’s correlations were performed to examine the associations between, HOMA-IR, hs-CRP, c-peptide, c-peptide: insulin ratio, SBP, DBP, HbA_1c_, and the TG:HDL ratio for the entire cohort. Partial correlations [i.e., controlling for fat mass (FM) and Tanner levels] and multivariate linear regression analyses [i.e., examination of the potential mediating role of the c-peptide: insulin ratio on the relationship between the predictor variable (hs-CRP) and the dependent variable (HOMA-IR)] and Sobel’s mediation test [33,34] were performed for each gender cohort. *p* <0.05 was considered significant.

## Results

### Findings stratified by gender

Table 1 summarizes the clinical and biochemical characteristics of the entire cohort as well as male and female subgroups. The males had higher BMI-SDS, FM and FFM than females (p < 0.05). While male adolescents had higher fasting glucose, HbA_1c_, insulin, HOMA-IR, hs-CRP, TG and TG:HDL-C ratio (p < 0.05) than females, the female adolescents had similar c-peptide levels, but had higher Tanner levels and c–peptide: insulin ratio than males (p < 0.001).

**Table 1:** Clinical and biochemical characteristics of participants based on gender

**Parameters**	**All**	**Males**	**Females**	***P***
N (%)	199	88 (44.2)	111 (55.8)	NA
Age (yrs)	15.5 ± 1.2	15.6 ± 1.1	15.5 ± 1.3	NS
Tanner stage	4.6 ± 0.5	4.5 ± 0.5	4.7 ± 0.4	<.0.001^d^
Race/ethnicity				
Caucasians (%)	87 (43.7)	31 (35.2)	56 (50.5)	NS
Hispanics (%)	60 (30.2)	31 (35.2)	29 (26.1)	NS
African Americans (%)	52 (26.1)	26 (29.6)	26 (23.4)	NS
SBP (mmHg)	127.7 ± 11.9	129.1 ± 11.1	126.6 ± 12.9	NS
DBP (mmHg)	69.1 ± 9.1	69.4 ± 9.2	68.8 ± 9.0	NS
BMI SDS	2.3 ± 0.4	2.5 ± 0.4	2.2 ± 0.3	<0.0001^d^
Fat (%)	43.8 ± 8.0	45.2 ± 9.8	42.7 ± 6.1	<0.05^a^
Fat Mass (kg)	47.1± 17.8	51.7 ± 20.2	43.4 ± 14.6	<0.001^c^
Fat-free Mass (kg)	57.8 ± 10.9	60.3 ± 13.7	55.9 ± 7.8	<0.01
FFM: FM Ratio	1.36 ± 0.4	1.31 ± 0.5	1.39 ± 0.3	NS
TBW (kg)	42.2 ± 8.0	43.9 ± 9.9	40.7 ± 5.7	NS
Glucose (mmol/L)	5.0 ± 0.5	5.1 ± 0.6	4.9 ± 0.4	<0.01^b^
HbA_1c_ (%)	5.3 ± 0.4	5.4 ± 0.5	5.2 ± 0.4	<0.01^b^
Insulin (pmol/L)	206.7 ± 92.2	244.2 ± 80.8	176.9 ± 90.2	<0.0001
HOMA-IR (mol uU/mL)	7.2 ± 3.7	8.1 ± 3.1	6.5 ± 3.4	<0.001^c^
c-peptide (pmol/L)	1302 ± 449.7	1337. 5 ± 430.5	1273.9 ± 464.3	NS
c-peptide: insulin ratio	7.1 ± 2.8	5.7 ± 1.6	8.1 ± 3.0	<0.0001^d^
hs-CRP (mg/L)	3.2 ± 1.4	5.4 ± 0.5	2.9 ± 1.2	<0.0001^d^
Triglycerides (mmol/L)	1.7 ± 0.9	1.9 ± 0.8	1.5 ± 0.9	<0.01^b^
Cholesterol (mmol/L)	4.6 ± 1.0	4.8 ± 0.9	4.5 ± 1.0	NS
HDL-C (mmol/L)	1.0 ± 0.2	1.1 ± 0.2	1.0 ± 0.2	NS
LDL-C (mmol/L)	2.9 ± 0.8	2.9 ± 0.8	2.8 ± 0.9	NS
Triglycerides: HDL-C ratio	4.0 ± 2.6	4.4 ± 2.6	3.6 ± 2.5	<0.05^a^
Prevalence of MS (%)	50.8	57.9	45.1	NS

Not applicable (NA) and not significant (NS).

^a^p < 0.05, ^b^p < 0.01, ^c^p < 0.001and ^d^p < 0.0001, for comparison of male vs. female group.

The AA subgroup displayed significantly higher BMI-SDS, FM and HbA_1c_ than H and C subgroups (data not shown, p < .0.01), whereas AA and H subgroups had higher insulin levels than C subgroup (data not shown, p < 0.01). In contrast, AA subgroup displayed significantly lower serum c-peptide and c-peptide: insulin ratio than H and C subgroups without racial/ethnic differences in hs-CRP and TG and TG: HDL-C ratio values (data not shown).

### Findings stratified by presence and absence of metabolic syndrome (MS)

While subjects with MS displayed higher BMI-SDS, FM, fasting blood glucose, HbA_1c_, insulin, c-peptide, HOMA-IR, hs-CRP, TG and TG: HDL-C ratio than the non-MS subjects (data not shown; p < .0.01), they displayed lower c-peptide: insulin ratio than the non-MS subjects (6.6 ± 2.7 vs. 7.4 ± 2.7, p < 0.05). Also, the prevalence of MS was similar among genders (M: 57.9% vs. F: 45.1%, p = NS) and racial/ethnic subgroups (C: 23.6%, H: 16.1% and AA: 11.1%; p = NS).

### Findings in the entire cohort

Table 2 provides a summary of the bivariate correlations among the clinical and biochemical variables for the entire cohort. Additional correlations between the clinical and biochemical variables and FM found that SBP, hs-CRP, HOMA-IR, TG: HDL-C, c-peptide and HbA_1c_ showed positive relationship with FM (p < 0.05), whereas c-peptide: insulin ratio displayed an inverse relationship with FM (p < 0.0001). While there were no gender differences in the relationship of FM with hs-CRP, HOMA-IR, TG: HDL-C ratio, c-peptide or the c-peptide: insulin ratio, there were gender differences in the relationship between FM and HbA1c [M: *r* = 0.36, *p* < 0.001) vs. F: *r* = 0.16, *p* = 0.10; *p* < 0.0001].

**Table 2:** Bivariate correlations for biomarkers of MS in the entire cohort

	**Fat mass (kg)**	**SBP (mmHg)**	**DBP (mmHg)**	**hsCRP (mg/L)**	**HOMA-IR (mol uU/mL)**	**TG: HDL-C ratio**	**c-peptide (pmol/L)**	**c-peptide: insulin ratio**	**HbA**_**1c **_**(%)**
**Fat mass (kg)**	1.0	0.139^a^	0.066	0.451^d^	0.516^d^	0.148^a^	0.393^d^	−0.332^d^	0.313^d^
**SBP (mmHg)**		1.0	0.464^d^	0.17^a^	0.076	0.053	−0.002	−0.121	0.119
**DBP (mmHg)**			1.0	0.073	0.087	0.074	0.082	−0.048	0.063
**hs-CRP (mg/mL)**				1.0	0.433^d^	0.368^d^	0.242^c^	−0.425^d^	0.296^d^
**HOMA-IR (mol uU/mL)**					1.0	0.307^d^	0.592^d^	−0.633^d^	0.370^d^
**TG: HDL ratio**						1.0	0.174^a^	−0.199^b^	0.166^a^
**c-peptide (pmol/L)**							1.0	0.074	0.105
**c-peptide: insulin ratio**								1.0	−0.365^d^
**HbA**_**1c **_**(%)**									1.0

^a^p < 0.05, ^b^p < 0.01, ^c^p < 0.001, ^d^p < 0.0001.

### Partial correlation findings in the male and female cohorts when controlling for FM and tanner stages

In order to determine whether FM and pubertal status was a potential mechanism through which gender influences the biomarkers of MS, subsequent partial correlation analyses, which controlled for FM and Tanner levels, were conducted for both males and females. Table 3 summarizes partial correlations among the clinical and biochemical variables for the entire cohort and both genders adjusted for FM and Tanner stage. Both hs-CRP and HOMA-IR showed positive relationships with the TG: HDL-C ratio, c-peptide and HbA1c in the entire cohort after adjusting for FM and Tanner stage (p < 0.01). In contrast, hs-CRP and HOMA-IR displayed negative relationships with c-peptide: insulin ratio (p < 0.01).

**Table 3:** Bivariate correlations for biomarkers of MS in the entire cohort and both genders adjusted for FM and Tanner stage

	**SBP (mmHg)**	**DBP (mmHg)**	**hs-CRP (mg/L)**	**HOMA-IR (mol uU/mL)**	**TG: HDL-C ratio**	**c-peptide (pmol/L)**	**c-peptide: insulin ratio**	**HbA**_**1c **_**(%)**
**SBP (mmHg)**								
Total	1.0	0.464^d^	0.170^a^	0.076	0.053	−0.002	−0.121	0.119
Male	1.0	0.444^d^	0.096	0.028	0.000	0.051	0.074	−0.003
Female	1.0	0.504^d^	0.097	−0.011	0.055	−0.133	−0.120	0.136
**DBP (mmHg)**								
Total		1.0	0.073	0.087	0.074	0.082	−0.048	0.063
Male		1.0	0.036	−0.049	−0.016	−0.020	−0.025	−0.010
Female		1.0	0.147	0.095	0.114	0.134	−0.010	0.058
**hs-CRP (mg/mL)**								
Total			1.0	0.433^d^	0.368^d^	0.240^c^	−0.425^d^	0.296^c^
Male			1.0	0.141	0.411^d^	0.147	0.113	0.035
Female			1.0	0.362^d^	0.497^d^	0.187	−0.309^c^	0.211^a^
**HOMA-IR (mol uU/mL)**								
Total				1.0	0.307^d^	0.592^d^	−0.633^d^	0.370^d^
Male				1.0	0.210	0.256^a^	−0.228^a^	0.008
Female				1.0	0.261^b^	0.670^d^	−0.616^d^	0.357^d^
**TG: HDL Ratio**								
Total					1.0	0.174^a^	−0.199^b^	0.166^a^
Male					1.0	0.113	0.022	0.130
Female					1.0	0.170	−0.151	0.067
**c-peptide (pmol/L)**								
Total						1.0	0.074.	0.105
Male						1.0	0.662^d^	−0.153
Female						1.0	0.084	0.127
**c-peptide: insulin Ratio**								
Total							1.0	−0.365^d^
Male							1.0	−0.169
Female							1.0	−0.297^b^
**HbA**_**1c **_**(%)**								
Total								1.0
Male								1.0
Female								1.0

^a^p < 0.05, ^b^p < 0.01, ^c^p < 0.001, ^d^p < 0.0001.

In males, only hs-CRP showed a significantly positive relationship with the TG: HDL-C ratio (p < 0.0001), but HOMA-IR had a negative relationship with c-peptide: insulin ratio (p < 0.0001). In females, hs-CRP displayed positive relationships with HOMA-IR, TG: HDL-C, c-peptide and HbA_1c_ (p < 0.05), whereas both hs-CRP and HOMA-IR showed inverse relationships with the c-peptide: insulin ratio (p < 0.001).

### Multivariate regression findings in the male and female cohorts

Multivariate linear regression analyses were conducted separately for both genders. The regression analyses examined the potential mediating role of the c-peptide: insulin ratio (index of insulin clearance) on the relationship between the predictor variable (hs-CRP) and the dependent variable (HOMA-IR), while controlling for FM. This relationship was only found to be significant among female participants (β = −0.54, t = −7.29, *p* < 0.001), but not for male participants. In order to determine the significance of this mediation model, the Sobel Test yielded a partial mediation model for female participants (β = 0.36, p < 0.001 to β = 0.18, p < 0.05; p < 0.01).

## Discussion

In the present study, hs-CRP and HOMA-IR were positively correlated in obese adolescents, but this relationship remained significant only in females after controlling for adiposity and pubertal status. We also found that the hs-CRP and HOMA-IR association was influenced by altered hepatic insulin clearance in females, especially after controlling for adiposity. Additional findings from the present study indicate that the c-peptide: insulin ratio was inversely correlated with hs-CRP and HOMA-IR and partially mediated the relationship between these biomarkers only among females. These findings indicate that there is a potential gender interaction for the association between HOMA-IR and hs-CRP. Specifically, sex hormones may play an important role in the inflammatory mechanism and development of insulin resistance.

Research has shown a strong association between IR and inflammation among adults [35], especially in obese non-diabetic patients with visceral adipose tissue [36]. Further, the relationship between IR and inflammation has been shown to have a stronger association in women than men [25]. Although there is minimal research in adolescents, the research thus far has found that adiposity is a strong determinant of inflammation among non-obese adolescents, with significant gender differences [37]. However, the quantity and distribution of FM influence inflammation to a greater extent in women compared to men [24]. In our study, hs-CRP was significantly correlated with FM in both male and female adolescents, without demonstrating any gender differences. Also, there were no gender differences in the prevalence of MS, but males had higher hs-CRP levels and HOMA-IR values than females, likely due to higher FM in males. However, positive relationship between hs-CRP and HOMA-IR for the entire cohort demonstrated a gender difference, where this relationship remained significant only in females after controlling for FM. These findings suggest that although adiposity plays a role in MS in both genders, female gender is associated with additional risk for MS independent of adiposity.

Chen et al. observed that fasting c-peptide was correlated with several markers of MS in obese adults and that basal c-peptide levels were significantly higher in women as compared to men [38]. Also, high fasting c-peptide level is a risk factor for atherosclerosis in both non-diabetic and diabetic adults [39,40] and correlated with components of MS in diabetic patients [39]. In our study, there were no gender differences in basal c-peptide levels, but c-peptide levels were significantly higher in MS than non-MS group. Basal c-peptide was positively correlated with FM and indices of inflammation (hs-CRP and TG: HDL-C) and IR for the entire cohort. Further, c-peptide remained positively correlated with HOMA-IR after adjusting for FM in the entire cohort and for both genders. However, the correlation between HOMA-IR and c-peptide was greater in female than male adolescents, suggesting a greater risk of atherosclerosis among females [41].

Decreased insulin clearance (c-peptide: insulin ratio) is a common finding among AA subjects compared to other racial/ethnic groups [42]. Additionally, it has been shown that several indices of MS, (i.e., TG, SBP and waist circumference) are associated with a decline of insulin clearance rate among adults [43]. Further, it has been shown that low grade inflammation induces insulin resistance through altered insulin signaling in hepatocytes and peripheral tissues [44]. Hepatic insulin clearance is positively related to insulin sensitivity and negatively to acute insulin response and adiposity across gender and race/ethnicity [45]. In our study, we found that AA subgroups had lower c-peptide: insulin ratios than C and H subgroups. Male adolescents and those with MS in the present study also demonstrated lower insulin clearance rate than female adolescents and non-MS subgroups, respectively. Insulin clearance was inversely correlated with indices of inflammation, IR and HbA_1c_ and this relationship remained after adjusting for FM. However, c-peptide: insulin ratio correlation with HOMA-IR was stronger among females than males, with insulin clearance partially mediating the relationship between hs-CRP and HOMA-IR only in females.

Limitations to this study include: retrospective design as well as lack of adiposity distribution data and oral glucose tolerance data to assess glucose homeostasis, beta-cell function and insulin clearance rate in relationship to hs-CRP and HOMA-IR. Also, the accuracy of bioelectrical impedance (BIA) for assessment of body composition has been questioned because of larger errors in individual estimates of body fat compared to DXA method [28]. However, BIA has been deemed accurate for assessing body composition in large groups of normal weight or obese pediatric subjects as compared to DXA [46]). Another limitation to the study is that there were no age- and sex-matched normal weight controls for each racial/ethnic group.

In conclusion, only female adolescents demonstrated an association between indices of inflammation and IR when adjusting for adiposity. This relationship between inflammation and IR was partially mediated by altered hepatic insulin clearance for female adolescents only. These findings imply that obese adolescent females may be at the greatest risk of developing early atherosclerosis and diabetes since decreasing insulin clearance inversely mediates the relationship between inflammation and insulin resistance independent of adiposity. Additional studies are needed to evaluate the relationship between chronic inflammation, IR, insulin clearance and development of vascular endothelial dysfunction in obese adolescents.

## Competing interest

The authors declare that they have no competing interests.

## Authors’ contributions

RA: collected and analyzed the data and wrote and edited the manuscript; JK: analyzed the data and wrote and edited the manuscript. Both authors read and approved the final manuscript.
